# Group B Streptococcal Colonization in African Countries: Prevalence, Capsular Serotypes, and Molecular Sequence Types

**DOI:** 10.3390/pathogens10121606

**Published:** 2021-12-10

**Authors:** Sarah Shabayek, Patricia Ferrieri, Barbara Spellerberg

**Affiliations:** 1 Department of Microbiology and Immunology, Faculty of Pharmacy, Suez Canal University, Ismailia 41522, Egypt; sarah.shabayek@pharm.suez.edu.eg; 2 Department of Laboratory Medicine and Pathology, University of Minnesota Medical School, Minneapolis, MN 55455, USA; ferri002@umn.edu; 3 Institute of Medical Microbiology and Hygiene, University Hospital Ulm, 89081 Ulm, Germany

**Keywords:** *Streptococcus agalactiae*, colonization, group B streptococci, neonatal infections, perinatal

## Abstract

*Streptococcus agalactiae* or group B streptococcus (GBS) is a commensal of the gastrointestinal and genitourinary tracts of healthy women and an important cause of neonatal invasive infections worldwide. Transmission of bacteria to the newborn occurs at birth and can be prevented by intrapartum antibiotic prophylaxis. However, this not available in resource limited settings in Africa, which carries a particular high burden of disease. Serotype based vaccines are in development and present a suitable alternative to prevent neonatal infections. To be able to assess vaccine efficacy, knowledge and surveillance of GBS epidemiological data are required. This review summarizes investigations about the serotype distribution and the multi-locus sequence types (MLST) found in different African countries. While most serotypes and MLST data are comparable to findings from other continents, some specific differences exist. Serotype V is predominant among colonizing maternal strains in many different African countries. Serotypes that are rarely detected in western industrialized nations, such as serotypes VI, VII and IX, are prevalent in studies from Ghana and Egypt. Moreover, some specific MLST sequence types that seem to be more or less unique to Africa have been detected. However, overall, the data confirm that a hexavalent vaccine can provide broad coverage for the African continent and that a protein vaccine could represent a promising alternative.

## 1. Introduction

*Streptococcus agalactiae* or group B Streptococcus (GBS), is part of the normal flora of the gastrointestinal and genitourinary tracts of healthy women. It is a leading cause of invasive disease in neonates such as sepsis, pneumonia, and meningitis [1,2]. GBS neonatal infections are classified as early-onset disease (EOD) which develops within 7 days after birth [3] and late-onset disease (LOD) presenting between days 7 and 90 after birth [4]. Maternal rectovaginal colonization is the major risk factor for transmission [1,2]. In addition, GBS can also cause serious infections in pregnant mothers, the elderly, and immunocompromised patients with underlying conditions [1,2,5,6]. The US Centers for Disease Control and Prevention (CDC) [7] recommended intrapartum antibiotic prophylaxis (IAP) for GBS positive carriers to prevent perinatal GBS disease. This is performed through a universal culture-based screening for pregnant women at 35–37 weeks of gestation. Active implementation of IAP has greatly reduced the incidence of GBS disease in the United States [7,8] and other industrialized countries, while it has not yet been adopted on the African continent.

GBS strains are classified by their capsular polysaccharides into ten serotypes: Ia, Ib, II-IX [9]. Further characterization, through multi-locus sequence typing (MLST), assigns strains to a defined sequence type (ST), based on allelic variation of seven housekeeping genes [10]. Closely related strains sharing several similar alleles of their STs are categorized into clonal complexes (CC) [10]. Molecular techniques, such as MLST and whole-genome sequencing (WGS), have shown that different capsular serotypes can have the same ST [11,12]. The relationship between serotypes, STs, and CCs are particularly important for serotype-based vaccines in the context of capsular switching and characterization of major neonatal virulent clones such as ST17. Such data add value beyond serotype alone [13,14]. The currently ongoing vaccine developments are mostly serotype-specific. Hence, their coverage will depend on the circulating serotypes [15,16]. GBS also possess structurally conserved immunogenic surface proteins that are crucial for GBS virulence [17,18]. The alpha-like protein (Alp) family are surface-anchored proteins including alpha-C, Rib, epsilon (Alp1), Alp2, Alp3, and Alp4 [18]. These are emerging as promising and cost-effective alternatives to serotype-based vaccines [17]. Clinical trials evaluating protein-based vaccines have been initiated [19]. Major limitations of GBS CPS-based vaccines include serotype dependence and capsular switching whereas polymorphisms in the sequences of the target protein antigen are important concerns for protein-based vaccines [14,20]. It is, therefore, critical to determine and monitor the global prevalence and serotype distribution of GBS isolates colonizing the rectovaginal tracts of pregnant women particularly in Africa where data are limited [14].

Until now, no licensed GBS vaccine is available although vaccine development was started over four decades ago [21]. Despite successful Phase 1 and Phase 2 clinical trials advancement to Phase 3 clinical trials testing vaccine efficacy has been postponed. Among the reasons for this postponement are the large number of patients required for a Phase 3 trial and the implementation of IAP as an effective prevention strategy for EOD [22] in industrialized countries. Furthermore, a continuous hesitancy to apply vaccines during pregnancy complicates a vaccine licensure primarily intended for maternal use [22]. However, IAP does not prevent LOD [14]. In addition, the true burden of GBS infections is larger than previously estimated and especially high in low-income settings [13,15]. The development of a GBS vaccine appropriate for maternal immunization of pregnant women in low and middle-income regions has recently been recognized as a priority by the World Health Organization (WHO) [14]. The health benefits of vaccinating pregnant women have been well-established and the availability of enhanced safety databases for maternal vaccination and the establishment of a precedent for immunizing pregnant women [22] greatly facilitate the development of a maternal vaccine. The aim of the current review is to shed light on predominant and region-specific serotype and MLST types of GBS in Africa that may have potential implications for future vaccine development.

## 2. GBS Prevalence and Serotype Predominance

A global meta-analysis by Russell et al., 2017 [16] estimated maternal prevalence of GBS colonization at 15% (95% confidence interval [CI], 14–16%) worldwide. The investigation detected similar figures in Africa ranging from 25% [95% CI, 22–29%]) in Southern Africa to 14% in Western Africa. This was later confirmed by a meta-analysis in Africa [23] where the highest GBS prevalence was estimated as 23.8% (95% CI 18.7, 28.9) in Southern African countries, followed by, 22.7% (95% CI 18.2, 27.2) in Northern African countries. The lowest maternal GBS prevalence was found in East Africa with a colonization rate of 15.4% (95% CI 12.1, 18.7) among pregnant women. These differences in GBS estimates of prevalence rates must be interpreted with caution because of study variations in sampling methods, diagnostic microbiology techniques, and study sizes.

Assessing the global situation, maternal GBS colonization with serotypes Ia, Ib, II, III, and V accounted for 98% of the detected serotypes [16]. However, serotype distribution demonstrated a limited variation over time from studies conducted pre-2001 to 2018 [13]. serotype IV was increasing in the developed regions of the Western world whereas serotypes Ia and Ib decreased in all other areas. For example, we demonstrated in Minnesota, USA the emergence of serotype IV over time [24]. Using whole genome sequencing it was shown that three sequence type 468 serotype IV strains isolated from neonates had acquired genes from a putative clonal complex 17 GBS donor by recombination [25]. Serotypes III, V and VI–IX had upward trends that could be observed in more recent investigations [13].

**Figure 1:**
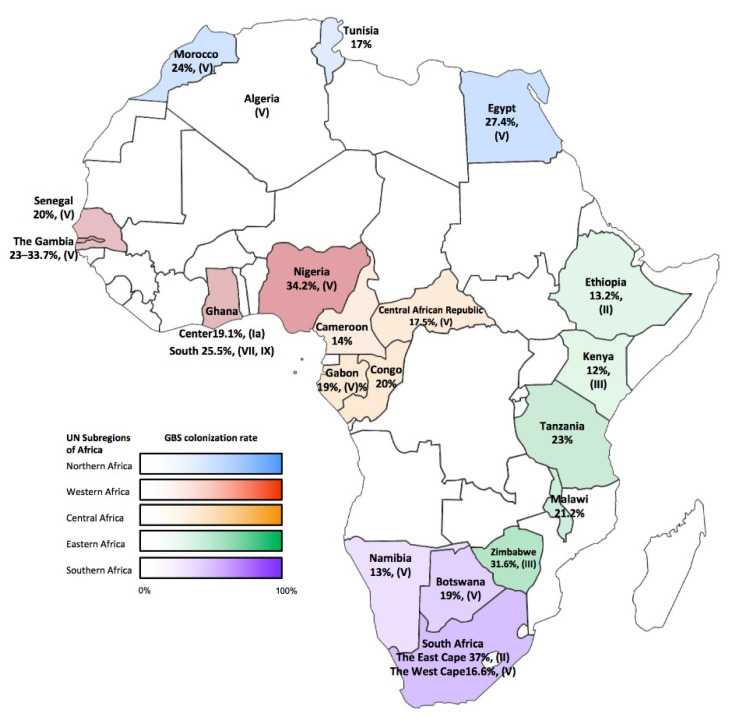
Depicted are GBS colonization rates and the main GBS serotype for countries, where data have been obtained. Shown are GBS colonization rates in % and as heatmaps. The main GBS serotypes found in each country are provided in parenthesis.

For Africa the picture is somewhat different, where serotypes V, III, Ia, Ib and II represent 91.8% of serotypes circulating among colonizing strains [23]. Epidemiological reports show serotype V is predominant in most regions of the African continent comprising not less than one third of the GBS isolates. This includes the North as reported in Egypt [26], Algeria [27] and Morocco [28]; in the West as shown in Senegal [29], Gambia [30,31] and Nigeria [32]; in central Africa as described in Gabon and Central African Republic [29]; the South as found in Botswana [33] and the Western Cape region of South Africa [34]. The reported prevalence ranged from 30.3 to 66.67%. serotype V also prevailed in Mozambique [13] and was common in central Ghana [35], Ethiopia [36], Zimbabwe [37,38,39] and Malawi [13]. The meta-analysis conducted by Gizachew et al., 2019 [23] showed serotype V accounting for 29.2% (95% CI 19.8, 38.6) of the GBS isolates as compiled from 14 African studies. GBS colonization rates and the main GBS serotypes for countries where data have been obtained are shown in Figure 1.

Despite the well-documented dominance of serotype V, regional variation in serotype distribution across the continent can still be seen. Intra-country variation in serotype predominance was also evident. Capsular Type II prevailed in both Namibia and the Eastern Cape region in South Africa [40]. The reported prevalence was 69% and 52.2% for serotype II versus 10.4% and 16.4% for serotype V, respectively. Capsular Type II is also the most often isolated serotype in Ethiopia [36,41,42]. The dominant serotype in coastal Kenya [43] and Malawi [13] was III constituting 38.3% and 38.9% of the isolates compared to 17% and 28.8% for serotype V, respectively. serotype III was also the most common serotype in Zimbabwe [37,38,39]. Moreover, the rare serotypes VII and IX predominated in southern Ghana [44] accounting for more than 60% of the GBS strains. In Egypt [26], the rare serotype VI exhibited a relatively high prevalence which was comparable to serotypes Ia, III and II. A meta-analysis on GBS serotypes in Africa demonstrated the pooled proportion of serotypes VI, VII, VIII and IX as 16%, 30%, 24.4% and 20.9% respectively [23], which is much higher than the rates of these serotypes found in western industrialized nations. serotype V represented 19% and 24% of maternal colonization in Europe and North America respectively whereas serotypes VI–IX represented less than 5% in Europe and North America [13].

## 3. The Predominant Multi-Locus Sequence Types

Multi-locus sequence typing (MLST) of GBS worldwide has clustered the isolates into six major clonal complexes (CC): CC1, CC10, CC17, CC19, CC23 and the rare CC26 [29,45]. The CCs 1, 10, 19 and 23 are generally well-adapted colonizers of the vaginal mucosa with low invasive potential. However, CC1 has been frequently reported in association with invasive infections in adults. Strains of CC17 especially serotype III are mostly associated with sepsis and meningitis in neonates [12,46]. For CC26, only Jones et al., 2006 [47] and Bohnsack et al., 2008 [48] reported occasional ST26 isolates in UK (3 out of 369) and the USA (2 out of 899). The worldwide diverse strain collection of Jones et al., 2003 [10] reported a single ST26 isolate from UK and 2 from Japan.

The CCs 1, 10, 17, 19, 23 were all reported in Africa in addition to CC26 which is rare in industrialized nations but found to be common in several African countries. Brochet et al., 2009 [29] showed 20% and 10.2% of GBS strains in Senegal and Central African Republic belonging to CC26, respectively. Of note, all these CC26 strains, except for one isolate in Central African Republic, were serotype V the predominate African serotype. However, this high percentage of CC26 strains in African studies was latter debated and claimed to be geographically restricted. Huber et al., 2011 [49] found only three isolates out of 169 in a strain collection from Kenya. In concordance, Seale et al., 2016 [43] found no CC26 strains out of 1036 in costal Kenya. It is noteworthy, that ST26 strains seem to be comparable to ST17 clones in regard to hypervirulence and may thus need more attention. MLST and complete genome sequencing revealed a hypervirulent non-typable isolate dating back to 1977 to be a ST26 strain with capsular Type V [50]. This strain, designated CNCTC 10/84, was originally isolated from human clinical samples [51] and has been widely used to investigate GBS pathogenesis due to its high virulence in animal models and overproduction of the beta-hemolysin [50].

Other MLST types with reported geographical restriction in Africa are ST484 and ST866 [12]. These lineages were rarely reported elsewhere. Seale and coworkers in 2016 [43] observed a large number of ST484 isolates in costal Kenya and all possessed the capsular Type III. The ST484 isolates were clustered in CC17 and represented 25% of the CC17 strains. An earlier study by Huber et al., 2011 [49] identified the same sequence type in Kenya as well. A pan-genome-wide association study (Pan-GWAS) found ST866 exclusively in Malawi [12]. The ST866 lineages also possessed the capsular Type III and clustered in CC17. Scanning the public MLST databases [52] revealed that STs 484 and 866 were not registered before except for a single ST484 isolate in Canada and two ST484 isolates in Belgium and Netherlands. However, ST866 was not registered elsewhere, indicating that all of these strains may primarily be of African origin.

Further geographical-dependent events were observed by Gori et al., 2020 [12] and Brochet et al., 2009 [29]. A particular cluster of genes including the gatKTEM system for galactose metabolism was present in a set of African lineages characterized as ST327 and ST328. This gene cluster was also associated with CC10. Gori et al., 2020 [12] proposed this association to be geographically dependent. Isolates of STs 327 and 328 are a subgroup of CC19. They associated with capsular Type V, instead of serotype III, which is more common in CC19 strains and these STs were rarely reported elsewhere. Table 1 shows MLST types with reported geographical restriction in Africa.

Taken together, although serotype V disseminates globally, it may be present in distinct genetic lineages in Africa as proposed by Brochet et al., 2009 [29]. Such strains may have evolved from a common ancestor of serotype V the most prevalent African serotype.

## 4. Associations between Multi-Locus Sequence Types and Serotypes

Literature indicates clustering of defined CCs with dominant capsular serotypes. Previously reported associations in the United States and Europe [11] were similar to those observed in Africa [12,27,29,32,41,42,49]. These associations included CC1 with serotype V, CC17 with serotype III and CC23 with serotype Ia [11]. Literature [12,27,29,32,41,42,49] and MLST database [43,52] for GBS records in Africa demonstrated dominant ST-serotype associations such as ST1 with serotype V, ST8 with serotype Ib, ST10 with serotype Ib and II, ST17 with serotype III, ST23 with serotype Ia, ST28 with serotype II, ST196 with serotype IV and finally STs 182 and 484 with serotype III. Similar trends were reported worldwide [11], indicating that while specific STs seem to be associated with African origin the association of MLST types with serotypes appears to be stable and irrespective of the strain origin.

## 5. Maternal Immunization

GBS is a leading cause of neonatal deaths in poor countries, with a high burden of illness in sub-Saharan Africa [15,53,54,55]. Intrapartum antibiotic prophylaxis (IAP) for colonized pregnant women has proven successful in reducing early-onset GBS disease in higher-income countries [7,8,15]. However, diagnosis of GBS colonization during pregnancy is not available in the vast majority of African settings due to a lack of resources and inadequate infrastructure. Thus, maternal screening and IAP are not feasible. This increases the risk of vertical GBS transmission at birth and, consequently, the propensity of developing GBS disease for the newborn. Empirical antibiotic therapy is the main practice and often occurs prior specimen collection. In addition, no prevention policies are available to reduce invasive diseases in neonates [15,56,57,58].

Sufficient knowledge on GBS epidemiology is lacking for most African regions. Information is most often restricted to capsular typing of GBS isolates from pregnant women and little data are available on invasive GBS disease in neonates and adults. The same is true for MLST data, which are scarce and only available for defined regions. Due to a lack of diagnostic possibilities within local healthcare settings and a general lack of health care accessibility in many regions, the burden of GBS infections is likely to be underestimated in most African countries. Despite the sparse data, regional estimates suggested that Africa may have a particularly high burden of invasive GBS neonatal infections [15,53,54,55]. The need for affordable interventions that are efficient in decreasing neonatal mortality is a paramount concern.

The CPS is a principal virulence factor in GBS [46,59]. Invasive GBS infections in neonates rely on the maternal antibody titer. Baker and Kasper (1976) [60] showed an inverse correlation between increased susceptibility of newborns to invasive GBS infections and the circulating serotype-specific capsular antibodies of their mothers. However, unconjugated CPS alone is poorly immunogenic [20,61]. Attention was drawn to conjugate vaccines following the success of the *Haemophilus influenzae* type b (Hib) vaccine [17,62]. Clinical studies proved CPS-protein conjugates to be highly immunogenic. Thus, GBS vaccine based on conjugating CPS to an immunogenic protein carrier is a well-studied vaccine approach [22,59]. Two protein conjugates are primarily used in GBS CPS-based vaccines: tetanus toxoid and the CRM197 diphtheria protein. Currently, the leading GBS vaccines in development are polysaccharide-protein conjugate vaccines and protein subunit vaccines [19]. GBS3 is a trivalent GBS CPS CRM197 conjugate vaccine comprising serotypes Ia, Ib, and III. While GBS3 completed Phase 2 clinical trials (Phase II: NCT02046148) [63], further development was halted due to limited disease coverage [22,64]. However, promising novel GBS vaccine candidates are currently being evaluated. These include GBS6 as a hexavalent CPS CRM197 conjugate vaccine covering serotypes Ia, Ib, II, III, IV, and V. It has already completed Phase 1/2 clinical trials (Phase I/II: NCT03170609, Phase II: NCT04258995) [22,65] and is waiting to be tested in a Phase 2B trial that will evaluate GBS6 administered concomitantly with tetanus, diphtheria, and acellular pertussis vaccine (Tdap) (NCT04766086). Another candidate, GBS-NN/NN2, that reached Phase 2 clinical trials (phase II: NCT04596878) [59] is a protein-based vaccine. It contains a combination of two alpha protein fusions: GBS-NN and GBS-NN2. GBS-NN comprises the highly immunogenic N-terminal domains of Alpha C and Rib proteins. GBS-NN2 is a fusion peptide derived from N-terminal domains of the other Alps.

Maternal immunization has been suggested as a cost-effective solution in low-income countries especially sub-Saharan Africa. The vaccine is expected to be cost-effective in countries with high case fatality ratio such as Ghana, Nigeria, and Guinea Bissau [15]. Despite regional variations in the reported serotype prevalence, GBS strains of serotypes Ia, Ib, II, III, and V are predominant in colonized women worldwide, including Africa, accounting for 98% of serotypes [16]. Seale and colleagues [43] predicted that a pentavalent vaccine would provide a 99% coverage of disease-causing serotypes in Africa and a trivalent vaccine including serotypes Ia, II, and III would be a good alternative. The vaccine would protect infants against both EOD and LOD, in contrast to IAP, which can only prevent EOD. A trivalent vaccine targeting women during pregnancy has already completed Phase II trials in several African countries [15] and further trials were postponed in order to develop a higher valency vaccine that would protect against almost all cases of infant disease [55]. Such a multivalent GBS vaccine is currently being evaluated in an ongoing trial in pregnant women in South Africa (NCT03765073). The primary purpose of this vaccine development program is to produce low-cost multivalent conjugate GBS vaccines with more than 90% coverage of invasive GBS disease in Africa [19]. However, the inclusion of particular serotypes should be considered. Seale and colleagues [43] previously recommended the inclusion of serotype IV due to evidence of capsular switching from serotype III in CC17 strains in Kenya. Studies in North America, using whole genome sequencing, revealed examples of single recombinatorial events leading to capsule switching [66]. Capsular switching occurred across multiple serotypes and among strains with dissimilar genomic backgrounds [66]. A global meta-analysis by Bianchi-Jassir and coworkers [13] reported 93–99% of GBS isolates were serotypes Ia, Ib, II, III, IV and V and accordingly recommended a hexavalent vaccine containing these serotypes in order to ensure broader coverage for all at-risk populations. serotype IV has now been included in the recent GBS6 vaccine [19,65]. The inclusion of region-specific serotypes should be considered as well for countries where minor serotypes predominate such as serotypes VII and IX in Ghana [44], or serotype VI in Egypt [26]. These vaccine approaches can capitalize on the development of a novel hexavalent (serotypes Ia, Ib, II, III, IV, and V) capsular polysaccharide GBS conjugate vaccine described recently for prevention of neonatal infections by maternal vaccination, and the Phase 1/2 trial in healthy non-pregnant adults [22,65].

## 6. Conclusions

GBS is a serious public health concern in Africa. Unfortunately, neither maternal screening nor IAP are available, since GBS diagnosis and adherence to IAP is difficult to implement in resource-poor African settings. As an alternative, maternal GBS immunization could be a cost-effective public health intervention providing protection against both forms of neonatal disease, EOD and LOD. It could have a positive impact on both maternal and neonatal morbidity and mortality [67].

Current vaccine efforts are based on GBS serotypes, therefore monitoring and knowledge of GBS epidemiology is crucial to ensure vaccine efficacy. Serotypes Ia, Ib, II, III, and V are the most predominant among colonized women, with serotype V being present in the majority of strains. While a pentavalent vaccine could already provide a broad coverage of relevant serotypes in Africa, the recently developed hexavalent GBS6 vaccine would ensure a full coverage of disease-causing serotypes ideally preventing all neonatal GBS infections.

## Figures and Tables

**Table 1:** MLST types with reported geographical restriction in Africa.

Clonal Complex (CC)	Sequence Type (ST)	Serotype	African Country	References
CC26	ST26	V	Senegal and Central African Republic	[29]
CC17	ST484	III	Costal Kenya	[43]
CC17	ST866	III	Malawi	[12]
CC19	ST327	V	Malawi and Kenya	[12]
CC19	ST328	V	Malawi and Kenya	[12]

## Data Availability

Data available in a publicly accessible repository that does not issue DOIs. Publicly available datasets were analyzed in this study. This data can be found here: [https://pubmlst.org/], accessed on 15 October 2021.
